# Preliminary Study on the Effect of Bronchial Epithelial Cell–Released Autophagosome (BA)–Induced Neutrophils on Bronchial Epithelial Cells

**DOI:** 10.1155/carj/9539112

**Published:** 2026-06-30

**Authors:** Rong Gao, Yuting Wu, Hongnian Lu, Zhifa Wen, Weizhen Qiao, Tingting Wang, Xiaoshan Li

**Affiliations:** ^1^ Department of Laboratory Medicine, The Affiliated Wuxi People’s Hospital of Nanjing Medical University, Wuxi People’s Hospital, Wuxi Medical Center, Nanjing Medical University, Wuxi, Jiangsu, 214023, China, njmu.edu.cn; ^2^ Nanjing Medical University, Nanjing, Jiangsu, 211166, China, njmu.edu.cn; ^3^ Department of Clinical Laboratory, Nanjing Women and Children’s Healthcare Hospital, Women’s Hospital of Nanjing Medical University, Nanjing, Jiangsu, 210004, China; ^4^ Department of Lung Transplantation Center, The Affiliated Wuxi People’s Hospital of Nanjing Medical University, Wuxi People’s Hospital, Wuxi Medical Center, Nanjing Medical University, Wuxi, Jiangsu, 214023, China, njmu.edu.cn

**Keywords:** asthma, bronchial epithelial cell apoptosis, bronchial epithelial cell–released autophagosomes, neutrophils

## Abstract

**Objective:**

To investigate the regulatory effects of bronchial epithelial cell–released autophagosome (BA)–induced neutrophils on bronchial epithelial cells and the underlying mechanisms.

**Methods:**

Transmission electron microscopy and western blotting were used to identify autophagosomes in 16HBE cells stimulated with house dust mite (HDM) antigen. The production of reactive oxygen species (ROS) and neutrophil extracellular traps (NETs) was detected after BA were cocultured with human peripheral blood neutrophils. Flow cytometry was used to detect the apoptosis rate of 16HBE cells incubated with cocultured supernatant, and BAX, Bcl‐2, and cleaved caspase 3 expression was detected by western blotting. In mouse acute asthma, hematoxylin and eosin (HE) staining was used to detect lung tissue injury. Apoptosis was detected by TUNEL, and neutrophils, macrophages, LC3, BAX, and Bcl‐2 were detected by immunohistochemistry.

**Results:**

HDM induced the release of autophagosomes by 16HBE cells. BA induced ROS production in neutrophils by activating NF‐κB signaling pathway and further promotes the formation of NETs, which induced apoptosis of 16HBE cells and was mediated by the regulation of BAX, Bcl‐2, and cleaved caspase 3. In the HDM‐induced mouse acute asthma, the lung tissue structure was destroyed, the content of neutrophils and macrophages increased, the macrophages showed M1 polarization, and the expression of LC3, BAX, Bcl‐2, and apoptosis increased.

**Conclusion:**

HDM‐stimulated 16HBE cells released autophagosomes, which induced ROS production in neutrophils via NF‐κB pathway activation and further promoted NET formation, ultimately leading to 16HBE cell apoptosis and lung injury in mice.

## 1. Background

Allergic asthma (AA) is the most prevalent type of asthma, and its prevalence has gradually increased in most parts of the world [1]. AA is usually a type I hypersensitivity reaction mediated by specific IgE, mainly via eosinophilic infiltration; however, approximately 20%–30% of global asthma cases are characterized by airway neutrophilic infiltration [2]. Patients with asthma and airway neutrophilic infiltration often present with severe clinical symptoms, and most of these patients are resistant to steroid therapy [3]. AA can be induced by various sensitizing substances, among which house dust mite (HDM) are the most common airway allergens. The clinical symptoms induced by HDM are relatively serious and prone to recurrence [4].

In AA cases, bronchial epithelial cells mediate the development of various inflammatory diseases by triggering autophagy. Many functions of neutrophils are related to autophagy, including cell differentiation, neutrophil extracellular traps (NETs) formation, and cytokine secretion and interaction [5]. A large number of autophagosomes have been detected in the bronchial fibroblasts and epithelial cells of patients with moderate and severe asthma [6]. And studies have found that inhibiting autophagy can limit mucoglycoprotein high expression and mucus production and reduce the production of eosinophils in the airways of mouse models of ovalbumin [7–9]. However, studies have shown that the absence of CD11c^+^ autophagy in lung tissue can lead to airway neutrophilic infiltration, thus inducing airway inflammation and resulting in airway hyperresponsiveness. Airway inflammation induced by autophagy deficiency is not sensitive to steroids [10]. The two‐sided effect of autophagy on airway inflammation makes it difficult to perform targeted immunotherapy against autophagy for the treatment of respiratory diseases.

Some studies have found that autophagosomes formed in yeast and mammalian cells, called secretory autophagosomes, can be released outside the cell, and secretory autophagosomes play a role in autophagy secretion as a nonclassical secretory mode [11, 12]. Compared to classical intracellular autophagosomes (known as degradative autophagosomes), secretory autophagosomes can avoid fusion with lysosomes and secrete cytoplasmic substrates [13–15]. Previous research from our group has shown that the secretory autophagosomes can be successfully recruited from various tumor cell culture supernatants or tumor chest/ascites specimens from patients with tumors by ultracentrifugation [16]. Autophagosomes and neutrophils, macrophages, and T cells are important in the tumor’s immune regulation. Tumor cell–released autophagosomes (TRAPs) have been shown to trigger macrophage polarization toward M2‐like phenotype via TRAPs–PD–L1 axis to drive immunosuppression in tumor microenvironment [17]. And the heat shock protein 90α on the surface of TRAPs programs the immunosuppressive functions of CD4^+^ T cells to promote tumor growth and metastasis [18]. However, there is still a lack of research on the regulation of immune function by autophagosomes released from bronchial epithelial cells in asthma.

Therefore, the present study starts from bronchial epithelial cell–released autophagosomes (BA) and neutrophils to explore whether HDM induces BA production and causes the neutrophil‐mediated effect on bronchial epithelial cells. By linking these two substances that play important roles in airway inflammation, the specific role of autophagy in airway inflammation was further explored, providing a theoretical basis for targeted autophagosome immunotherapy. Moreover, HDM nasal drops were used to induce sensitization in mice, and a mouse model of acute AA was constructed. Animal experiments were conducted to verify the possible immune responses in the airways under HDM sensitization.

## 2. Materials and Methods

### 2.1. Cell Lines and Culture Conditions

The bronchial epithelial cell line, 16HBE, was preserved aseptically in the laboratory at −80°C. 16HBE cells were resuscitated using DMEM medium containing 10% FBS and 1% penicillin–streptomycin solution (100 ×) and cultured in a 37°C and 5% CO_2_ incubator. Cells were passaged once every 3 days.

### 2.2. Mice

Specific pathogen‐free‐grade male Balb/c mice aged 6–8 weeks and weighing 18–20 g were purchased from Changzhou Cavens Laboratory Animal Co., Ltd. The feeding environment was maintained at 20°C–26°C, humidity 50%–60%, ammonia concentration ≤ 14 mg/m^3^, and the feeding density was 5 mice per cage. All animal experiments were approved by the Nanjing Medical University Experimental Animal Welfare Ethics Committee and conducted in accordance with the ethical guidelines.

### 2.3. Acute AA Mouse Model

Ten male BALB/c mice were included in the model. The acute AA model group (*n* = 5) was sensitized by 25 μg of HDM (414,145, GREER) i.n. on Days 1, 2, and 3 and stimulated using 25 μg of HDM i.n. daily from Days 8 to 15 [19]. The control group (*n* = 5) was stimulated with an equal volume of 1 × PBS. 24 h after the final administration of HDM, anesthesia was administered intraperitoneally with 0.3% pentobarbital sodium at 50 mg/kg, and peripheral blood and lung tissue samples were obtained.

### 2.4. Autophagosome Extraction

16HBE cells were cultured for 24 h and then stimulated with 2 μg/mL [20] of HDM. Following stimulation, the medium was replaced. After 24 h, the cells were collected, digested with an appropriate amount of pancreatic enzymes, and centrifuged at 1000 rpm for 5 min, and the supernatant was collected. The cell medium and supernatant were centrifuged at 12,000 rpm for 15 min. The supernatant was discarded, and the precipitate was washed once with PBS and centrifuged at 12,000 rpm for 15 min. The main components of the resulting precipitation were BA, which were dissolved in PBS and frozen at −80°C for later use. The total protein concentration was measured using a BCA Protein Assay Kit (WB6501, NCM Biotech) according to the manufacturer’s instructions, and LC3‐II content was verified by western blotting.

### 2.5. Transmission Electron Microscopy

The newly obtained autophagosome precipitates were fixed using an electron microscope fixation solution and 1% osmic acid. The samples were dehydrated in increasing concentrations of alcohol, permeabilized with acetone and 812 coating agent, and then sliced. The samples were stained with 2% uranium acetate and lead citrate, their morphology and structure were detected by transmission electron microscopy, and transmission electron microscopy images were collected.

### 2.6. Isolation and Purification of Human Peripheral Blood Neutrophils

The cell separation solutions Histopaque‐1077 (10,771, Sigma), Histopaque‐1119 (11,191, Sigma), and whole blood were successively added to centrifuge tubes. After centrifugation at 700 g for 30 min, the cells between the two separation fluids in the centrifuge tube were neutrophils. After the red blood cells were lysed, the neutrophils were resuspended in complete medium with PMI‐1640 containing 10% FBS and 1% penicillin–streptomycin solution (100 ×).

### 2.7. Flow Cytometry

16HBE cells were prepared as single‐cell suspensions, and the apoptosis ratio of 16HBE cells was determined using the Apoptosis Detection Kit (KGA1030‐50, KeyGEN BioTECH) according to the manufacturer’s instructions. Neutrophils from human peripheral blood were isolated and purified. The reactive oxygen species (ROS) content in neutrophils was determined using an ROS Detection Kit (S0033S, Beyotime) according to the manufacturer’s instructions. Deionized water repeatedly lysed mouse peripheral blood red blood cells to obtain white blood cells. The following antimouse antibodies (eBioscience) were used: PERCPCYN5.5‐CD45, FITC‐CD3, APC‐CD4, PE‐CD8, APC‐EP780‐CD11B, PE‐CYN7‐LY6G, APC‐F4‐80, PE‐CD206, and FITC‐MHC II.

### 2.8. Enzyme‐Linked Immunosorbent Assay (ELISA)

The concentration of NETs produced by neutrophils in human peripheral blood was detected using an MPO‐DNA ELISA Kit (23,452, MEIMIAN), according to the manufacturer’s instructions.

### 2.9. Immunofluorescence

Human peripheral blood neutrophils were fixed with 4% PFA and permeabilized with a mixture of 0.3% Triton X‐100 and 5% BSA. Diluted antimyeloperoxidase (MPO) (1:200) and antihistone‐H3 (1:100) were added and incubated overnight at 4°C. Fluorescent antibodies 594 and 488 were added and incubated in the dark at room temperature for 1 h and then incubated with 2 μg/mL of DAPI in the dark at room temperature for 15 min. The slides were sealed with glycerin, observed under a fluorescence microscope, and images were recorded.

### 2.10. Western Blotting

Cells were lysed in NCM RIPA buffer containing protease and phosphatase inhibitors. The samples were resolved by SDS‐PAGE and transferred to PVDF membrane after blocking. Then, the membranes were incubated with primary antibodies overnight at 4°C and exposed to secondary antibodies. Protein bands were visualized using a fully automatic chemiluminescent gel analyzer. Anti‐LC3I, anti‐LC3II, anti‐caspase 3/p17/p19, anti‐BAX, anti‐Bcl2, anti‐p65, anti‐p‐p65, and anti‐beta actin were used as primary antibodies, and goat antimouse IgG HRP was used as a secondary antibody.

### 2.11. HE Staining of Mouse Lung Tissue Samples

Mouse lung sections were dewaxed with xylene and alcohol and stained with hematoxylin and eosin. The samples were dehydrated with xylene and alcohol and sealed with neutral gum. Microscopic observation, image acquisition, and analysis were performed.

### 2.12. Immunohistochemistry of Mouse Lung Tissue Samples

Mouse lung sections were dewaxed with xylene and alcohol and repaired with citric acid antigen repair buffer. BSA was added to block endogenous peroxidase. The primary antibody was added and incubated at 4°C overnight, and the fluorescent secondary antibody was added and incubated at room temperature without light for 50 min. Nuclei were stained with a DAPI dye solution. The slices were sealed with an antifluorescence quenching sealant and observed under a fluorescence microscope. Images were collected and analyzed.

### 2.13. Statistical Analysis

Data were analyzed using GraphPad Prism 9.5.0 software. A paired *t*‐test was used to compare the two groups (the normal‐distribution test was performed before the paired *t*‐test). A bidirectional analysis of variance was used for intragroup comparisons. One‐way analysis of variance was used to compare multiple groups. Each experiment was independently performed at least three times unless otherwise stated. Differences were considered statistically significant at *p* < 0.05. Significant differences are shown in figures with asterisks (^∗^ *p* < 0.05, ^∗∗^ *p* < 0.01, ^∗∗∗^ *p* < 0.001, and ^∗∗∗∗^ *p* < 0.0001).

## 3. Results

### 3.1. HDM Stimulates Bronchial Epithelial Cells 16HBE Cells to Release BA Outside the Cell

**FIGURE 1:**
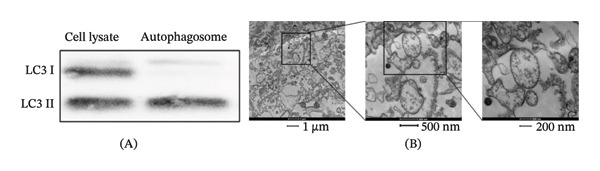
Bronchial epithelial 16HBE cells release autophagosomes into the extracellular space. (A) LC3I and LC3II levels in 16HBE cell sediments and cell lysate were detected by western blotting, and LC3II level was high in the sediments. (B) The biological morphology of the precipitates was detected by transmission electron microscopy. Vesicle structures with double‐layer membranes varied in size (200–1000 nm in diameter).

HDM stimulates 16HBE cells to release BA outside the cell. The LC3I and LC3II were detected using western blotting, and the morphology and structure of the sediments were observed using transmission electron microscopy. Western blotting results showed that LC3II levels were high (Figure 1A). Transmission electron microscopy revealed vesicle structures with double‐layer membranes varying in size (approximately 200–1000 nm in diameter; Figure 1B). These results demonstrated that bronchial epithelial 16HBE cells can release autophagosomes into the extracellular space.

### 3.2. Autophagosome‐Induced Neutrophils Promoted Apoptosis of Bronchial Epithelial Cells by Regulating BAX, Bcl2, and Cleaved Caspase3 Expression

**FIGURE 2:**
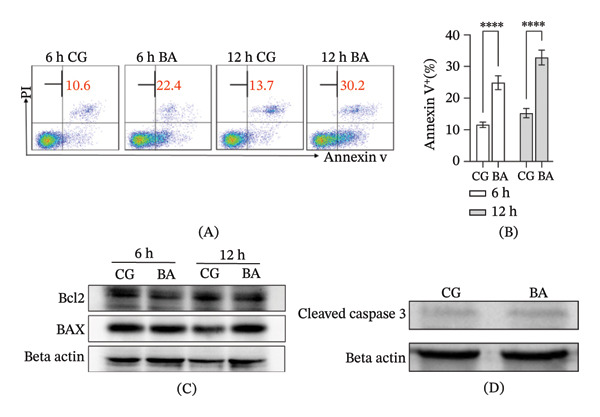
BA‐induced neutrophils promote 16HBE cell apoptosis by enhancing BAX and cleaved Caspase3 expression and inhibiting Bcl2 expression. (A and B) 16HBE cells were cultured with neutrophil supernatant treated with BA for 6 and 12 h, and the apoptosis ratio of 16HBE cells was detected by flow cytometry. (C) 16HBE cells were cultured with neutrophil supernatant treated with BA for 6 and 12 h, and the expressions of Bcl2 and BAX in 16HBE cells were detected by western blotting. (D) 16HBE cells were cultured with BA‐treated neutrophil supernatant, and the expression of cleaved Caspase3 in 16HBE cells was detected by western blotting.

16HBE cells released BA upon HDM stimulation. To detect the effect of coculture of BA and neutrophils on bronchial epithelial cells, 10 μg/mL of BA was cocultured with human peripheral blood neutrophils. After 6 and 12 h, the cell supernatant was collected to culture 16HBE cells. The apoptosis ratio of 16HBE cells was detected using flow cytometry, and western blotting was used to detect the expression of the apoptosis‐related proteins BAX, Bcl2, and Cleaved Caspase3 in 16HBE cells. Compared with the control group, the proportion of apoptotic 16HBE cells increased at 6 and 12 h in the BA group (Figure 2A,B). The results showed that the expression of the proapoptotic proteins BAX and Cleaved Caspase3 was increased in group BA, while the expression of the antiapoptotic protein Bcl2 was decreased (Figure 2C,D). These results suggest that BA‐induced neutrophils promote 16HBE cell apoptosis by enhancing BAX and Cleaved Caspase3 and inhibiting Bcl2.

### 3.3. BA Promotes 16HBE Cell Apoptosis by Stimulating ROS Production via Neutrophils

**FIGURE 3:**
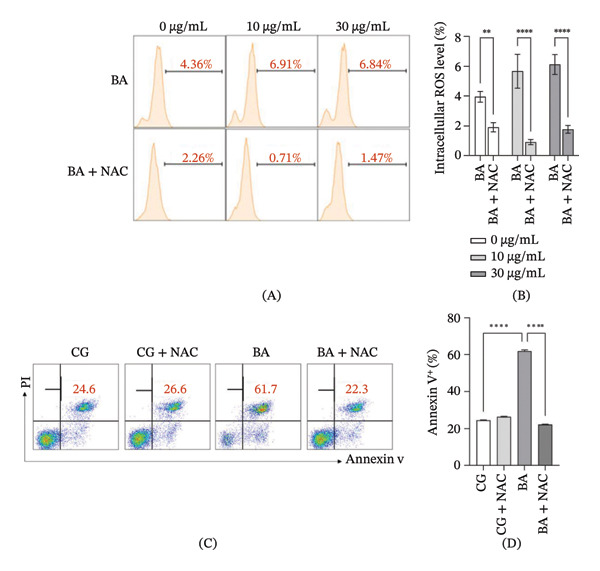
BA stimulated neutrophils to produce ROS, which promoted 16HBE cell apoptosis. (A and B) Neutrophils alone and neutrophils pretreated with 7.5 mmol/L of NAC for 30 min were incubated, respectively, with 0, 10, and 30 μg/mL of BA for 30 min, and their ROS content was determined by flow cytometry. (C and D) Neutrophils were cocultured with 10 μg/mL of BA for 6 h, and the supernatant was collected. The supernatant alone and the supernatant pretreated with 7.5 mmol/L of NAC for 2 h were coincubated with 16HBE, and the apoptosis ratio of 16HBE cells was detected by flow cytometry.

To explore the mechanism by which BA‐induced neutrophils promote apoptosis of 16HBE cells, we detected whether BA stimulated neutrophils to release ROS by flow cytometry and used N‐acetyl‐L‐cysteine (NAC) to eliminate the effect of ROS. Then, the effect of BA‐induced neutrophils on 16HBE cell apoptosis was detected. Human peripheral blood neutrophils were incubated with 0, 10, and 30 μg/mL of BA for 30 min or pretreated with 7.5 mmol/L of NAC for 30 min. Then, they were incubated with 0, 10, and 30 μg/mL of BA for 30 min, and the ROS content was detected by flow cytometry. The results showed that BA stimulated neutrophils to produce ROS. As BA concentration increased, there was no significant change in the production of ROS, whereas NAC neutralized ROS (Figure 3A,B). After human peripheral blood neutrophils were cocultured with 10 μg/mL of BA for 6 h, cell supernatant was collected, or the supernatant was pretreated with 7.5 mmol/L of NAC for 2 h, and the supernatant was coincubated with 16HBE. Apoptosis in 16HBE cells was detected using flow cytometry. The results showed that NAC reduced the proportion of apoptotic 16HBE cells cultured with the neutrophil supernatant induced by BA (Figure 3C,D). These results suggest that BA promotes apoptosis of 16HBE cells by stimulating the production of ROS by neutrophils.

### 3.4. BA‐Stimulated Neutrophil ROS Production, Promoted NETs Production, and Induced Apoptosis of 16HBE Cells

**FIGURE 4:**
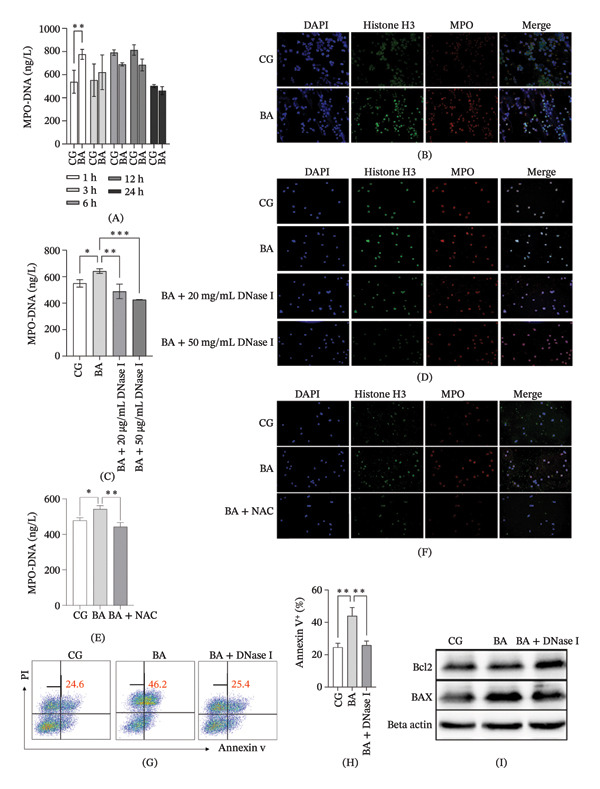
BA stimulated neutrophil ROS production, promoted NET production, and induced apoptosis of 16HBE cells. (A) Human peripheral blood neutrophils were cocultured with 10 μg/mL of BA for 1, 3, 6, 12, and 24 h. (B) MPO and histone H3 expression in human peripheral blood neutrophils cocultured with 10 μg/mL of BA for 1 h was detected by immunofluorescence (40 ×). (C) After human peripheral blood neutrophils were cocultured with 10 μg/mL of BA for 1 h, the cell supernatant was pretreated with different concentrations of DNase I (0, 20, 50 μg/mL) for 30 min, and the concentration of MPO‐DNA in the supernatant was detected by ELISA. (D) Human peripheral blood neutrophils were cocultured with 10 μg/mL of BA for 1 h, treated with different concentrations of DNase I (0, 20, 50 μg/mL) for 30 min, and the expression of MPO and histone H3 were detected by immunofluorescence (40 ×). (E) Human peripheral blood neutrophils were pretreated with 7.5 mmol/L of NAC for 30 min and then were incubated with 10 μg/mL of BA for 1 h. The concentration of MPO‐DNA in the supernatant was detected by ELISA. (F) Human peripheral blood neutrophils were pretreated with 7.5 mmol/L of NAC for 30 min and then were incubated with 10 μg/mL of BA for 1 h. The expression of MPO and histone H3 was detected by immunofluorescence (40 ×). (G and H). After human peripheral blood neutrophils were cocultured with 10 μg/mL of BA for 1 h, the supernatant was collected, and the supernatant was treated with 50 μg/mL of DNase I for 30 min. 16HBE cells were cultured, and the apoptosis ratio of 16HBE cells was detected by flow cytometry. (I) After human peripheral blood neutrophils were cocultured with 10 μg/mL of BA for 1 h, the supernatant was collected, and the supernatant was treated with 50 μg/mL of DNase I for 30 min, and 16HBE cells were cultured, and the expressions of autophagy‐related proteins BAX and Bcl2 were determined by western blotting.

To investigate the mechanism by which BA‐induced neutrophils promote the apoptosis of 16HBE cells, we determined whether BA stimulated neutrophils to release NETs using ELISA and immunofluorescence. After neutralizing NETs with deoxynucleotidase I (DNase I), 16HBE cell apoptosis was detected using flow cytometry. Human peripheral blood neutrophils were cocultured with 10 μg/mL of BA for 1, 3, 6, 12, and 24 h, and the cell supernatant was collected. The MPO‐DNA concentration was determined using ELISA. The results showed that the concentration of NETs in the neutrophil supernatant after BA stimulation was significantly higher than that in the control group at 1 h. Over time, the concentrations of NETs in the BA and control groups showed no significant differences and gradually decreased (Figure 4A). After human peripheral blood neutrophils were cocultured with 10 μg/mL of BA for 1 h, the expressions of NETs‐specific markers MPO and histone H3 were detected by immunofluorescence. The results showed that the fluorescence intensity of MPO and histone H3 in the BA group was significantly enhanced compared to that in the control group (Figure 4B). Human peripheral blood neutrophils were cocultured with 10 μg/mL of BA for 1 h, and cell supernatants were collected and pretreated with 0, 20, and 50 μg/mL of DNase I for 30 min. The concentration of supernatant MPO‐DNA was detected by ELISA, and the expressions of NETs‐specific markers MPO and histone H3 were detected by immunofluorescence. The results showed that the concentration of MPO‐DNA, fluorescence intensity of MPO, and histone H3 gradually decreased as DNase I concentration increased (Figure 4C,D). To investigate the interaction between ROS and NETs after BA stimulation. Human peripheral blood neutrophils were pretreated with 7.5 mmol/L of NAC for 30 min and then were incubated with 10 μg/mL of BA for 1 h. Supernatants and cells were collected to detect NETs content by ELISA and immunofluorescence. The results showed that NAC reduced the production of NETs (Figure 4E,F), suggesting that ROS can promote the production of NETs. After human peripheral blood neutrophils were cocultured with 10 μg/mL of BA for 1 h, the cell supernatant was collected, the supernatant was pretreated with 50 μg/mL of DNase I for 30 min, and 16HBE cells were cultured. Flow cytometry was used to detect the apoptosis ratio of 16HBE cells, and western blotting was used to detect the expression of apoptosis‐related proteins BAX and Bcl2. The results showed that the apoptotic rate of 16HBE cells cultured in neutrophil supernatants induced by BA decreased after DNase I treatment (Figure 4G,H). The results showed that, compared with the control group, the expression of the proapoptotic protein BAX was increased, and the expression of the antiapoptotic protein Bcl‐2 was decreased in the BA group. Compared to the BA group, the expression of the proapoptotic protein BAX decreased, and the expression of the antiapoptotic protein Bcl‐2 increased in the BA + DNase I group (Figure 4I). These results suggest that BA stimulates neutrophils to produce NETs, enhances BAX, and inhibits Bcl‐2, which promotes 16HBE cell apoptosis.

### 3.5. BA Induces ROS Production in Neutrophils by Activating NF‐κB Signaling Pathway and Further Promotes the Formation of NETs

**FIGURE 5:**
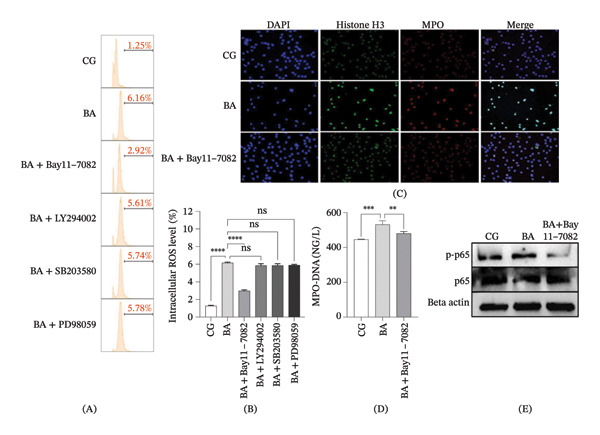
BA induces ROS production in neutrophils by activating NF‐κB signaling pathway and further promotes the formation of NETs. (A and B) Human peripheral blood neutrophils pretreated with Bay11‐7082 (3 μM), LY294002 (5 μM), SB203580 (10 μM), and PD98059 (10 μM) for 30 min and then were incubated with 10 μg/mL of BA for 1 h, and ROS content was determined by flow cytometry. (C) Human peripheral blood neutrophils pretreated with 3 μM, Bay11‐7082 for 30 min and then were incubated with 10 μg/mL of BA for 1 h. The expression of MPO and histone H3 was detected by immunofluorescence (40 ×). (D) Human peripheral blood neutrophils pretreated with 3 μM, Bay11‐7082 for 30 min and then were incubated with 10 μg/mL of BA for 1 h. The concentration of MPO‐DNA in the supernatant was detected by ELISA. (E) Human peripheral blood neutrophils pretreated with 3 μM, Bay11‐7082 for 1 h and then were incubated with 10 μg/mL of BA for 2 h, and the expressions of autophagy‐related proteins p65 and p‐p65 were determined by western blotting.

To investigate how BA stimulation drives ROS and NETs production in neutrophils at the molecular level. After treatment with the inhibitors of signaling pathways NF‐κB (Bay11‐7082, 3 μM), PI3K/Akt (LY294002, 5 μM), and ERK/MAPK (SB203580, 10 μM and PD98059, 10 μM), the production of ROS was detected. Inhibitor Bay11‐7082 reduced ROS content, but there was no significant change in the ROS content after inhibitors LY294002, SB203580, and PD98059 treatment (Figure 5A,B). Human peripheral blood neutrophils were pretreated with Bay11‐7082 for 30 min and then were incubated with 10 μg/mL of BA for 1 h. Supernatants and cells were collected to detect NETs. ELISA and immunofluorescence results showed that the inhibitor Bay11‐7082 reduced the production of NETs (Figure 5C,D). The results of western blotting showed that BA promotes the phosphorylation of p65, a key protein in the NF‐κB pathway in neutrophils, and this effect can be inhibited by Bay11‐7082 (see Figure 5E). These results suggest that BA induces ROS production in neutrophils by activating NF‐κB signaling pathway and further promotes the formation of NETs.

### 3.6. Lung Tissue Injury and Autophagy Level Were Increased in HDM‐Induced Acute AA Mouse Model

**FIGURE 6:**
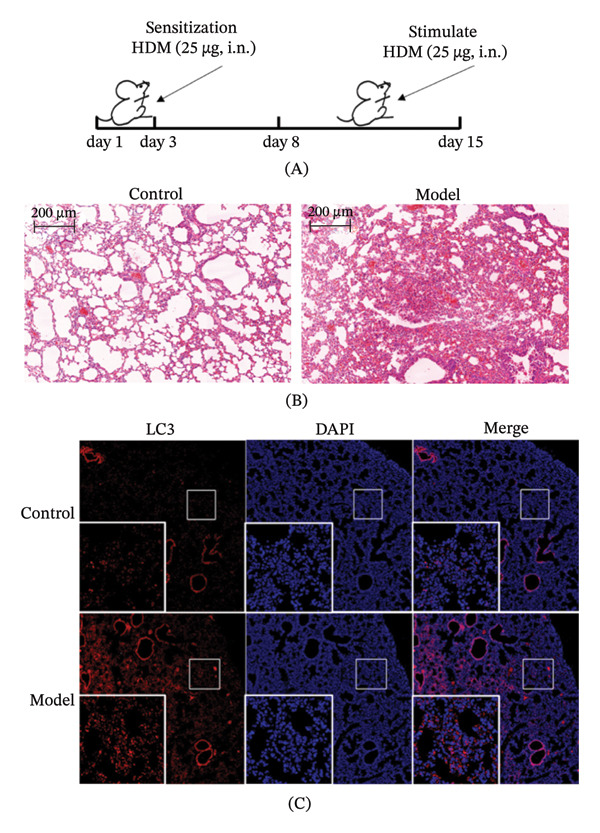
HDM‐induced AA mouse. (A) Balb/c male mice were divided into model and control groups. On Days 1, 2, and 3, 25 μg of HDM nasal drops were administered for sensitization, and on Days 8–15, 25 μg of HDM i.n. were administered daily. Peripheral blood and lung specimens were harvested on Day 16, and the control group was stimulated with equal volume of 1 × PBS. (B) HE staining of paraffin sections of lung tissue. (C) LC3 expression in mouse lung tissues was detected by immunofluorescence in paraffin sections of lung tissues (5 × and 40 ×).

BALB/c mice were stimulated with HDM nasal drops to establish an AA model (Figure 6A). The pathological results of HE staining of paraffin sections of lung tissue showed that the alveolar structures in mice were damaged, and alveolar collapse and extrusion, inflammatory cell infiltration, and the alveolar wall thickening were observed, and the boundary was unclear. In the control group, the lung tissue structure was complete, alveolar walls were thin, distribution was orderly, and boundaries were clear (Figure 6B). To explore the level of autophagy in the lung tissues of mice with HDM‐induced AA, LC3 expression in mouse lung tissues was detected by immunofluorescence. The results showed that the fluorescence intensity of LC3 in the lung tissues of model group mice was higher than that in control group mice (Figure 6C). These results indicated that lung tissue injury was aggravated in the HDM‐induced AA model, and the expression level of autophagy‐related proteins was increased, suggesting that autophagy might occur in lung tissue under HDM stimulation.

### 3.7. Elevated Neutrophils in Peripheral Blood and Lung Tissue of HDM‐Induced AA Mouse Model

**FIGURE 7:**
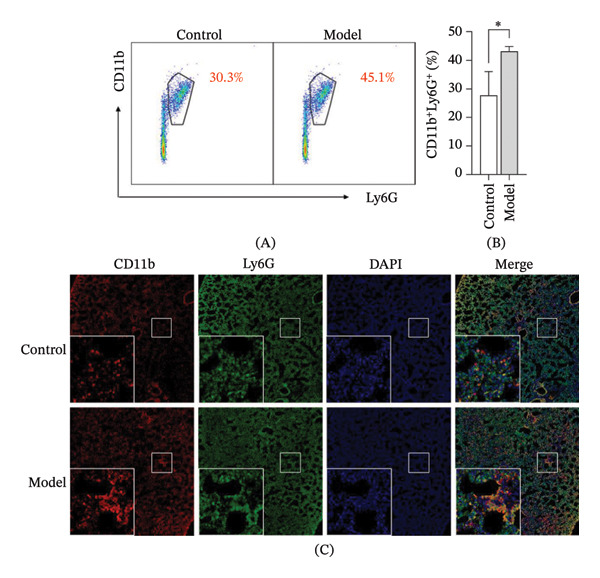
Neutrophil levels in peripheral blood and lung tissue were increased in the AA model group. (A and B) The proportion of neutrophils in peripheral blood of mice was determined by flow cytometry. (C) CD11b and Ly6G levels in paraffin sections of lung tissue were determined by immunofluorescence (5 × and 40 ×).

To investigate the number of neutrophils in the HDM‐induced AA model, the proportion of neutrophils in the peripheral blood of mice was measured by flow cytometry. The results showed that the proportion of neutrophils in the peripheral blood of model group mice was significantly higher than that in control group mice (Figure 7A,B). The expression of neutrophil‐specific cytokines CD11b and Ly6G in the lung tissues of mice was detected by immunofluorescence, and the fluorescence intensity of CD11b and Ly6G in the lung tissues of model group mice was higher than that in control group mice (Figure 7C). These results indicate that neutrophils were elevated in the peripheral blood and lung tissue of mice with HDM‐induced AA. Simultaneously, flow cytometry was used to detect the proportions of CD4^+^ and CD8^+^ T cells in the peripheral blood of mice to explore whether immune T cells participated in the immune process of lung tissue damage in the model group. The results showed that the proportion of CD4^+^ T cells in the peripheral blood of mice in the model group increased, the proportion of CD8^+^ T cells decreased, and the proportion of CD4^+^/CD8^+^ T cells increased (Supporting Figure A–D).

### 3.8. Mouse Lung Tissue Cells Promote Apoptosis by Regulating the Expression of Apoptosis‐Related Proteins, Leading to Lung Tissue Injury

**FIGURE 8:**
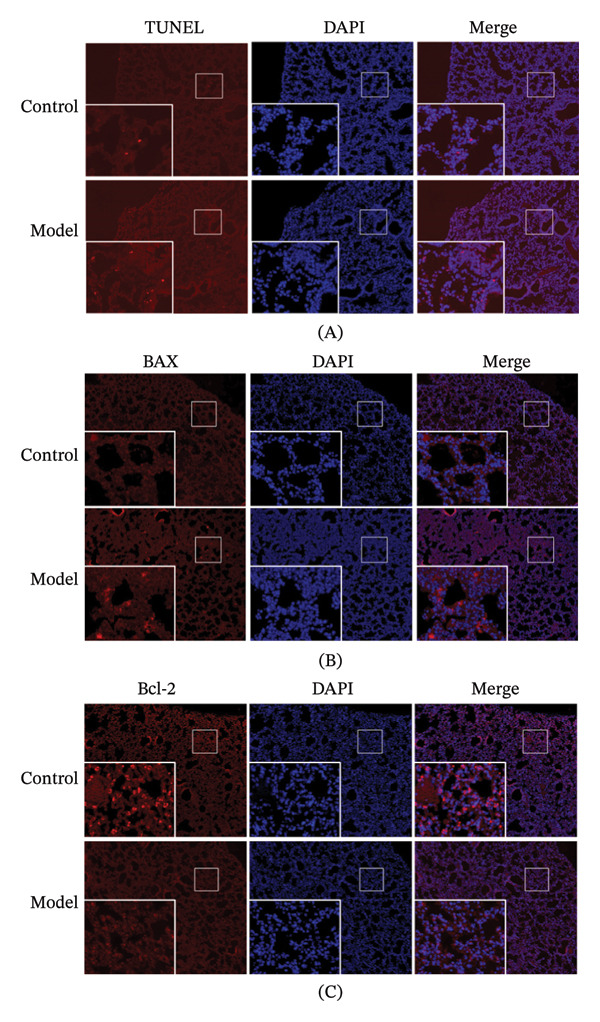
After HDM stimulation, mouse lung tissue cells promote apoptosis by regulating the expression of apoptosis‐related proteins. (A) TUNEL staining was used to detect apoptosis in mouse lung tissue (5 × and 40 ×). (B and C) The expressions of apoptosis‐related proteins BAX and Bcl‐2 in mouse lung tissue were detected by immunohistochemistry (5 × and 40 ×).

To investigate the mechanism of lung tissue injury in the model group, apoptosis was detected by TUNEL staining, and the apoptosis‐related proteins BAX and Bcl‐2 were detected by immunohistochemistry. The results showed that the fluorescence intensity of TUNEL staining, and the associated proportion of apoptotic lung tissue cells, in the model group was higher than that in the control group (Figure 8A). The fluorescence intensity of the proapoptotic protein BAX in the model group was higher than that in the control group (Figure 8B), indicating that the expression of the proapoptotic protein BAX in the lung tissue cells of mice in the acute asthma model group was higher. The fluorescence intensity of the antiapoptotic protein Bcl‐2 in the model group was lower than that in the control group (Figure 8C), indicating that the expression of the proapoptotic protein Bcl‐2 in the lung cells of mice in the acute asthma model group was lower. These results suggest that after HDM stimulation, mouse lung tissue cells undergo apoptosis by regulating the expression of apoptosis‐related proteins, leading to lung tissue injury.

### 3.9. HDM Induced AA Model Mice Lung Tissue Recruitment of Macrophages and Promote Macrophage M1 Polarization to Aggravate Inflammatory Response

**FIGURE 9:**
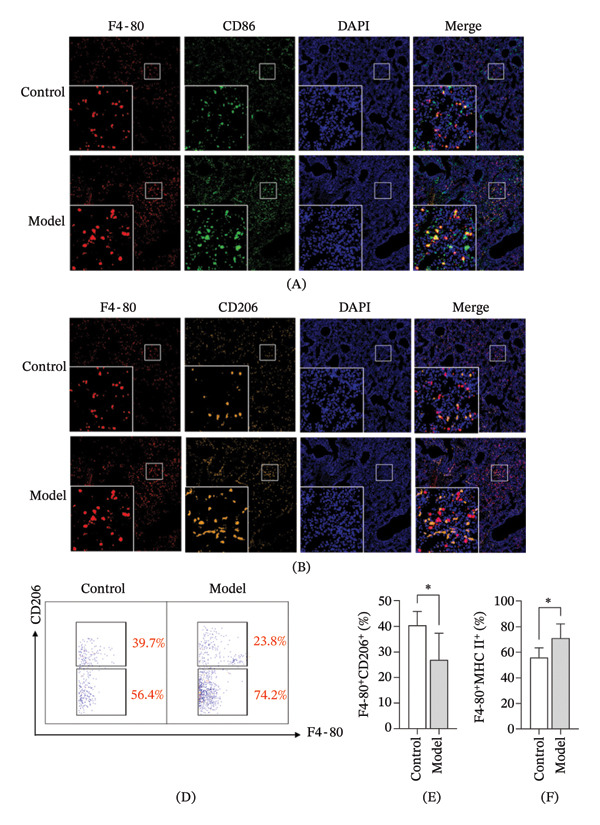
HDM stimulation increased the proportion of macrophages in mouse lung tissue and promoted M1 polarization of macrophages. (A and B) F4‐80, CD86, and CD206 in paraffin sections of lung tissue were determined by immunofluorescence (5 × and 40 ×). (C–E) Flow cytometry was used to detect the proportion of CD206^+^ macrophages and MHC II^+^ macrophages in mouse lung tissues.

To investigate the role of macrophages in the lung tissue of acute asthma model group, F4‐80, CD86, and CD206 were detected by immunohistochemistry, and the proportion of CD206^+^ macrophages and MHC II^+^ macrophages in the lung tissue of mice was detected by flow cytometry. The results showed that the fluorescence intensity of F4‐80 in model group was higher than that in control group (Figure 9A,B), indicating macrophage recruitment in the lung tissue of AA model group mice. The fluorescence intensity of F4‐80 and CD86 colocalization in the model group was higher than that in the control group (Figure 8A), while the fluorescence intensity of F4‐80 and CD206 colocalization in the model group was not significantly different from that in the control group (Figure 9B). The content of F4‐80^+^ MHC II^+^ macrophages in the lung tissue was higher than that in F4‐80^+^ CD206^+^ (Figure 9C–E). It indicated that the macrophages in the lung tissue of the AA model group were mainly M1‐type macrophages. These results suggest that HDM induced AA model mice lung tissue recruitment of macrophages and promote macrophage M1 polarization to aggravate inflammatory response.

## 4. Discussion

In an inflammatory environment, neutrophils are recruited and activated by related cytokines, and the autophagy process is also triggered. Activated neutrophils induce epithelial cell injury and increase mucus production by releasing neutrophilic elastase, MPO, ROS, and other substances [21]. Cell‐derived ROS are mainly released by inflammatory cells such as eosinophils, neutrophils, and macrophages in the lungs. Excessive production of ROS or the impairment of antioxidant reactions leads to an imbalance of reoxidation and reduction, called oxidative stress, resulting in tissue damage and dysregulation of cell transduction signals, thus inducing a variety of diseases, including asthma [22]. NETs are network structures composed of neutrophil DNA, histone H3, and MPO. Although NETs are involved in pathogen elimination, they also have cytotoxic effects on bronchial epithelium, thus exacerbating asthma and other airway diseases [23, 24]. Our study suggests that in asthma patients sensitized to HDM allergens, the occurrence of autophagy and the recruitment of neutrophils in the airways may promote the release of ROS, triggering an oxidative stress response. This further facilitates the formation of NETs, ultimately mediating the initiation and progression of airway inflammation.

Numerous studies have demonstrated that neutrophils can release ROS through the activation of multiple signaling pathways, thereby participating in inflammatory responses. In both acute pancreatitis and gastric cancer, neutrophils can be regulated via the NF‐κB–HIF‐1α pathway and thereby participate in immune responses [25, 26]. Meanwhile, NET formation aggravates renal ischemia–reperfusion injury by activating the ST2/PI3K/Akt pathway [27]. And moreover, MEK/ERK/PKC and JNK signaling pathways induce ROS generation, leading to NET formation through azurosome complex–mediated neutrophil elastase release [28]. In this experiment, ROS production by neutrophils stimulated by BA was inhibited only by inhibitors of the NF‐κB signaling pathway, which may be due to different cell models and stimulation or different time periods of inhibitors. The experimental results of different cell models and different time points can be further explored in future studies.

The caspase family includes critical enzymes that trigger apoptosis, and activation of these proteases eventually causes cells to enter the irreversible apoptosis process. Cleaved Caspase3 is often used as a marker protein for detecting apoptosis [29]. Studies have found that the antiapoptotic protein Bcl‐2 can inhibit proapoptotic members of the Bcl2 family and inhibit Beclin‐1 protein from interfering with autophagy, and high levels of Bcl‐2 can cause autophagy‐associated protein‐5 to lose its proapoptotic function [30]. BAX and Bcl‐2 antagonists are proapoptotic proteins that activate the intrinsic apoptotic pathway [31]. The results indicate that the ROS and NETs produced by BA‐stimulated neutrophils directly affect the expression of apoptosis‐related proteins in 16HBE cells and lead to apoptosis in 16HBE cells. But the specific mechanism by which this occurs needs to be further explored.

HDM stimulated 16HBE cells to release autophagosomes; therefore, we selected HDM as an allergen to develop a mouse asthma model. At the cellular level, we demonstrated that HDM could promote the apoptosis of 16HBE cells after stimulating BA release by 16HBE cells and interacting with neutrophils. However, in addition to bronchial epithelial cell damage, a series of inflammatory reactions occur during bronchial asthma. Therefore, we stimulated airway modeling with HDM to explore the possible expression of autophagy‐related proteins and generation of inflammatory cells in mice under HDM stimulation and determine their effects on lung tissue structure.

Studies have shown that the activation of CD4^+^T cells precedes that of CD8^+^T cells because of their interaction with migratory dendritic cells and that CD4^+^T cells are crucial in the formation of memory‐protective CD8^+^T cells after infection or immunization [32, 33]. In our experimental model, the increased proportion of CD4^+^T cells indicates that there is already an immune response in the airway. We speculate that the proportion of CD8^+^T cells will gradually increase with the increase of sensitization time, which needs further experiments. Moreover, the number of neutrophils increased in the peripheral blood and lung tissues, which is consistent with the basic characteristics of airway inflammation. Because neutrophil infiltration in the airways of asthmatic patients is often associated with more severe clinical symptoms [34], we believe that asthma triggered by HDM as an allergen may be characterized by clinical severity and refractory. Macrophages play crucial roles in maintaining homeostasis in the lung microenvironment and exhibit both proinflammatory and anti‐inflammatory functions in respiratory inflammatory diseases such as AA [35]. Modulation of macrophage polarization may be a potential therapy for these diseases [36]. HDM‐induced AA model mice also showed macrophage infiltration and M1 polarization in lung tissue, which was involved in the development of inflammatory response. These results suggest that there are pathophysiological mechanisms involving multiple immune cells and immune responses in AA model mice.

At the same time, animal experiments showed that under HDM stimulation, the body may induce asthma by initiating autophagy and inflammation, eventually causing lung tissue cell apoptosis and lung tissue structure breakdown. Consistent with the results of the cellular experiments, apoptosis of lung tissue and bronchial epithelial cells was accompanied by an increase in BAX and a decrease in Bcl‐2. These experimental results provide a theoretical basis for autophagosome‐targeted therapy for bronchial asthma. However, owing to the limitations of the experimental conditions, the lack of determination and analysis of lung function in experimental mice is one of the shortcomings of this study. The specific mechanisms and pathways by which BA promotes the apoptosis of bronchial epithelial cells and induces lung autophagy and apoptosis by HDM remain to be studied.

## 5. Conclusions

This study demonstrated that HDM stimulates 16HBE cells to release autophagosomes outside the cell. BA induces ROS production in neutrophils by activating the NF‐κB signaling pathway and further promotes the formation of NETs and apoptosis of 16HBE cells. Apoptosis in bronchial epithelial cells is mediated by changes in the expression of BAX, Bcl‐2, and cleaved Caspase3 in bronchial epithelial cells. Through animal experiments, we found that HDM stimulation increased the number of neutrophils and CD4^+^ immune T cells in the peripheral blood of mice, that neutrophils and macrophages infiltrated the lung tissue of mice, the macrophages showed M1 polarization, and that the expression of autophagy‐related proteins increased. HDM also stimulated an increase in BAX and a decrease in Bcl‐2 in lung tissue cells, which promoted apoptosis of lung tissue cells and destroyed the lung tissue structure. These results provide a theoretical basis for blocking autophagy in bronchial epithelial cells as a therapeutic strategy to reduce the clinical symptoms of patients with asthma.

## Funding

The study was supported by funding from the NSFC (82101875), the General Program of Wuxi Medical Center, Nanjing Medical University (WMCG202339), and the Young Medical Talents of Wuxi People’s Hospital (GR) (2024‐YZ‐QNBJ‐GR‐2024).

## Ethics Statement

This study protocol was reviewed and approved by the Nanjing Medical University Experimental Animal Welfare Ethics Committee, approval number SYXK2020‐0010.

## Conflicts of Interest

The authors declare no conflicts of interest.

## Supporting Information

Additional supporting information can be found online in the Supporting Information section.

## Supporting information


**Supporting Information 1** The ARRIVE guidelines 2.0: author checklist.

**Supporting Information 2** Supporting Figure. CD4^+^ T cell increases in mice with HDM‐induced AA. A–D, Flow cytometry was used to detect the proportion of CD4^+^ T cells and CD8^+^ T cells in the peripheral blood of mice.

## Data Availability

The data that support the findings of this study are available from the corresponding authors upon reasonable request.

## References

[1] Eguiluz-Gracia I. , Palomares F. , Salas M. et al., Precision Medicine in House Dust Mite-Driven Allergic Asthma, Journal of Clinical Medicine. (2020) 9, no. 12, 10.3390/jcm9123827. PMC7761474 33255966

[2] Crisford H. , Sapey E. , Rogers G. B. et al., Neutrophils in Asthma: The Good, the Bad and the Bacteria, Thorax. (2021) 76, no. 8, 835–844, 10.1136/thoraxjnl-2020-215986. 33632765 PMC8311087

[3] Moore W. C. , Hastie A. T. , Li X. et al., Sputum Neutrophil Counts are Associated With More Severe Asthma Phenotypes Using Cluster Analysis, Journal of Allergy and Clinical Immunology. (2014) 133, no. 6, 1557–1563, 10.1016/j.jaci.2013.10.011. 24332216 PMC4040309

[4] Ruggieri S. , Drago G. , Longo V. et al., Sensitization to Dust Mite Defines Different Phenotypes of Asthma: A Multicenter Study, Pediatric Allergy & Immunology. (2017) 28, no. 7, 675–682, 10.1111/pai.12768. 28783215

[5] Painter J. D. , Galle-Treger L. , and Akbari O. , Role of Autophagy in Lung Inflammation, Frontiers in Immunology. (2020) 11, 10.3389/fimmu.2020.01337. PMC7358431 32733448

[6] Poon A. H. , Chouiali F. , Tse S. M. et al., Genetic and Histologic Evidence for Autophagy in Asthma Pathogenesis, Journal of Allergy and Clinical Immunology. (2012) 129, no. 2, 569–571, 10.1016/j.jaci.2011.09.035. 22040902 PMC3268897

[7] Silveira J. S. , Antunes G. L. , Kaiber D. B. et al., Autophagy Induces Eosinophil Extracellular Traps Formation and Allergic Airway Inflammation in a Murine Asthma Model, Journal of Cellular Physiology. (2020) 235, no. 1, 267–280, 10.1002/jcp.28966. 31206674

[8] Zhang Y. , Tang H. , Yuan X. et al., TGF-β3 Promotes MUC5AC Hyper-Expression by Modulating Autophagy Pathway in Airway Epithelium, EBioMedicine. (2018) 33, 242–252, 10.1016/j.ebiom.2018.06.032. 29997053 PMC6085582

[9] Liu J. N. , Suh D. H. , Trinh H. K. , Chwae Y. J. , Park H. S. , and Shin Y. S. , The Role of Autophagy in Allergic Inflammation: A New Target for Severe Asthma, Experimental and Molecular Medicine. (2016) 48, no. 7, 10.1038/emm.2016.38. PMC4973311 27364893

[10] Suzuki Y. , Maazi H. , Sankaranarayanan I. et al., Lack of Autophagy Induces Steroid-Resistant Airway Inflammation, Journal of Allergy and Clinical Immunology. (2016) 137, no. 5, 1382–1389, 10.1016/j.jaci.2015.09.033. 26589586 PMC4860134

[11] Pillay J. , Tak T. , Kamp V. M. , and Koenderman L. , Immune Suppression by Neutrophils and Granulocytic Myeloid-Derived Suppressor Cells: Similarities and Differences, Cellular and Molecular Life Sciences. (2013) 70, no. 20, 3813–3827, 10.1007/s00018-013-1286-4. 23423530 PMC3781313

[12] Zea A. H. , Rodriguez P. C. , Atkins M. B. et al., Arginase-Producing Myeloid Suppressor Cells in Renal Cell Carcinoma Patients: A Mechanism of Tumor Evasion, Cancer Research. (2005) 65, 3044–3048, 10.1158/0008-5472.CAN-04-4505. 15833831

[13] Minakaki G. , Menges S. , Kittel A. et al., Autophagy Inhibition Promotes SNCA/Alpha-Synuclein Release and Transfer via Extracellular Vesicles With a Hybrid Autophagosome-Exosome-Like Phenotype, Autophagy. (2018) 14, no. 1, 98–119, 10.1080/15548627.2017.1395992. 29198173 PMC5846507

[14] Ponpuak M. , Mandell M. A. , Kimura T. , Chauhan S. , Cleyrat C. , and Deretic V. , Secretory Autophagy, Current Opinion in Cell Biology. (2015) 35, 106–116, 10.1016/j.ceb.2015.04.016. 25988755 PMC4529791

[15] Jiang S. , Dupont N. , Castillo E. F. , and Deretic V. , Secretory Versus Degradative Autophagy: Unconventional Secretion of Inflammatory Mediators, Journal of Innate Immunity. (2013) 5, 471–479, 10.1159/000346707. 23445716 PMC3723810

[16] Gao R. , Ma J. , Wen Z. et al., Tumor Cell-Released Autophagosomes (TRAP) Enhance Apoptosis and Immunosuppressive Functions of Neutrophils, Oncoimmunology. (2018) 7, no. 6, 10.1080/2162402X.2018.1438108. PMC5980412 29872581

[17] Wen Z. , Liu H. , Gao R. et al., Tumor Cell-Released Autophagosomes (TRAPS) Promote Immunosuppression Through Induction of M2-Like Macrophages With Increased Expression of PD-l1, Journal for Immunotherapy of Cancer. (2018) 6, no. 1, 10.1186/s40425-018-0452-5. PMC6299637 30563569

[18] Chen Y. , Li P. , Pan N. et al., Tumor-Released Autophagosomes Induces CD4(+) T Cell-Mediated Immunosuppression via a TLR2-IL-6 Cascade, Journal for Immunotherapy of Cancer. (2019) 7, no. 1, 10.1186/s40425-019-0646-5. PMC6625067 31300052

[19] Brüggemann T. R. , Carlo T. , Krishnamoorthy N. et al., Mouse Phospholipid Phosphatase 6 Regulates Dendritic Cell Cholesterol, Macropinocytosis, and Allergen Sensitization, iScience. (2022) 25, no. 10, 10.1016/j.isci.2022.105185. PMC9550614 36238896

[20] Vroling A. B. , Jonker M. J. , Luiten S. , Breit T. M. , Fokkens W. J. , and van Drunen C. M. , Primary Nasal Epithelium Exposed to House Dust Mite Extract Shows Activated Expression in Allergic Individuals, American Journal of Respiratory Cell and Molecular Biology. (2008) 38, no. 3, 293–299, 10.1165/rcmb.2007-0278OC. 17901406

[21] Hammad H. and Lambrecht B. N. , The Basic Immunology of Asthma, Cell. (2021) 184, no. 6, 1469–1485, 10.1016/j.cell.2021.02.016. 33711259

[22] Michaeloudes C. , Abubakar-Waziri H. , Lakhdar R. et al., Molecular Mechanisms of Oxidative Stress in Asthma, Molecular Aspects of Medicine. (2022) 85, 10.1016/j.mam.2021.101026. 34625291

[23] Balloy V. and Chignard M. , The Innate Immune Response to *Aspergillus fumigatus* , Microbes and Infection. (2009) 11, no. 12, 919–927, 10.1016/j.micinf.2009.07.002. 19615460

[24] Dworski R. , Simon H. U. , Hoskins A. , and Yousefi S. , Eosinophil and Neutrophil Extracellular DNA Traps in Human Allergic Asthmatic Airways, Journal of Allergy and Clinical Immunology. (2011) 127, no. 5, 1260–1266, 10.1016/j.jaci.2010.12.1103. 21315435 PMC3085562

[25] Wang Q. , Zhang X. , Han C. et al., Immunodynamic Axis of Fibroblast-Driven Neutrophil Infiltration in Acute Pancreatitis: Nf-Κb-Hif-1Α-Cxcl1, Cellular and Molecular Biology Letters. (2025) 30, no. 1, 10.1186/s11658-025-00734-6. PMC12060353 40335899

[26] Zhang X. , Shi H. , Yuan X. , Jiang P. , Qian H. , and Xu W. , Tumor-Derived Exosomes Induce N2 Polarization of Neutrophils to Promote Gastric Cancer Cell Migration, Molecular Cancer. (2018) 17, no. 1, 10.1186/s12943-018-0898-6. PMC6174070 30292233

[27] Zhang F. , Wu J. , Li Z. et al., Interleukin-33 Promotes Neutrophil Extracellular Trap Formation to Aggravate Renal Ischemia-Reperfusion Injury Through ST2/PI3k/akt and ST2/PAD4 Pathways, Inflammation. (2026) 49, no. 1, 10.1007/s10753-025-02364-8. PMC12882866 41557019

[28] Wang H. , Kim S. J. , Lei Y. et al., Neutrophil Extracellular Traps in Homeostasis and Disease, Signal Transduction and Targeted Therapy. (2024) 9, no. 1, 10.1038/s41392-024-01933-x. PMC11415080 39300084

[29] Tonnus W. , Meyer C. , Paliege A. et al., The Pathological Features of Regulated Necrosis, Journal of Pathology. (2019) 247, no. 5, 697–707, 10.1002/path.5248. 30714148

[30] Horn J. , Stelzner K. , Rudel T. , and Fraunholz M. , Inside Job: *Staphylococcus aureus* Host-Pathogen Interactions, International Journal of Medical Microbiology. (2018) 308, no. 6, 607–624, 10.1016/j.ijmm.2017.11.009. 29217333

[31] Ahsan N. , Shariq M. , Surolia A. , Raj R. , Khan M. F. , and Kumar P. , Multipronged Regulation of Autophagy and Apoptosis: Emerging Role of TRIM Proteins, Cellular and Molecular Biology Letters. (2024) 29, no. 1, 10.1186/s11658-023-00528-8. PMC10790450 38225560

[32] Laidlaw B. J. , Craft J. E. , and Kaech S. M. , The Multifaceted Role of CD4(+) T Cells in CD8(+) T Cell Memory, Nature Reviews Immunology. (2016) 16, no. 2, 102–111, 10.1038/nri.2015.10. PMC4860014 26781939

[33] Hor J. L. , Whitney P. G. , Zaid A. , Brooks A. G. , Heath W. R. , and Mueller S. N. , Spatiotemporally Distinct Interactions With Dendritic Cell Subsets Facilitates CD4^+^ and CD8^+^ T Cell Activation to Localized Viral Infection, Immunity. (2015) 43, no. 3, 554–565, 10.1016/j.immuni.2015.07.020. 26297566

[34] Chen F. , Yu M. , Zhong Y. , Hua W. , and Huang H. , The Role of Neutrophils in Asthma, Zhejiang Da Xue Xue Bao Yi Xue Ban. (2021) 50, no. 1, 123–130, 10.3724/zdxbyxb-2021-0030. 34117844 PMC8675072

[35] Chen S. , Saeed A. F. U. H. , Liu Q. et al., Macrophages in Immunoregulation and Therapeutics, Signal Transduction and Targeted Therapy. (2023) 8, no. 1, 10.1038/s41392-023-01452-1. PMC10200802 37211559

[36] Li B. , Xia C. , He W. et al., The Thyroid Hormone Analog GC-1 Mitigates Acute Lung Injury by Inhibiting M1 Macrophage Polarization, Advanced Science. (2024) 11, no. 44, 10.1002/advs.202401931. PMC11600256 39373388

