# Supplementary material for: Preliminary Study on the Effect of Bronchial Epithelial Cell–Released Autophagosome (BA)–Induced Neutrophils on Bronchial Epithelial Cells
Source: Can Respir J. 2026 Jun 30;2026:9539112. doi: 10.1155/carj/9539112 (PMC13317461; doi:10.1155/carj/9539112)
Supplement: Supplementary file 2 — Supporting Information 2 Supporting Figure. CD4+ T cell increases in mice with HDM‐induced AA. A–D, Flow cytometry was used to detect the proportion of CD4+ T cells and CD8+ T cells in the peripheral blood of mice. [file CARJ-2026-9539112-s002.docx]

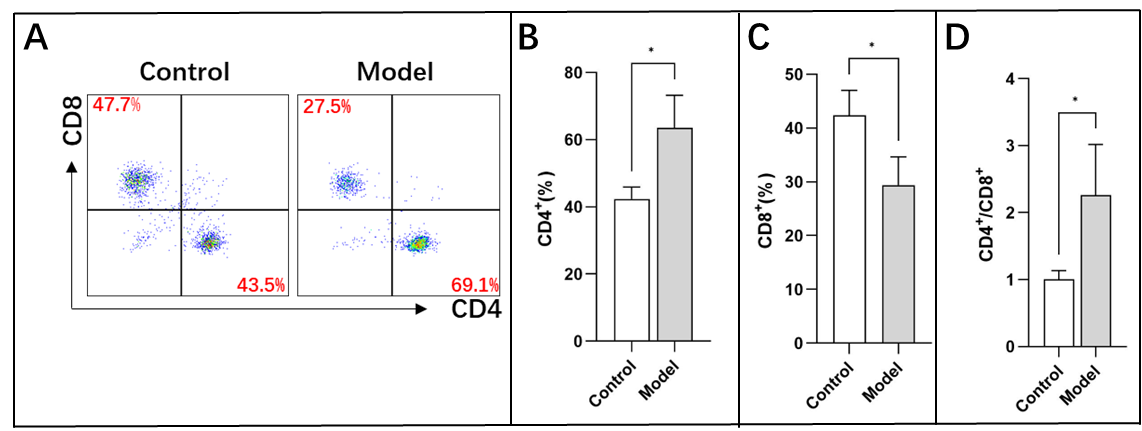


**Supplementary Figure**. CD4^+^ T cell increase in mice with HDM-induced AA. **A-D,** Flow cytometry was used to detect the proportion of CD4^+^ T cells and CD8^+^ T cells in the peripheral blood of mice.
